# Gaining comprehensive data about sexual knowledge through surveys

**Published:** 2017-04-10

**Authors:** Zahra Karimian, Effat Merghati Khoei, Raziyeh Maasoumi, Marzieh Araban, Mahboobeh Rasoulzadeh Bidgoli, Shahrokh Aghayan, Seied Ali Azin

**Affiliations:** 1 *Department of Midwifery, faculty of Nursing and Midwifery, Kashan University of Medical Sciences, Kashan, Iran.*; 2 *The Iranian National Center for Addiction Studies (INCAS), Institution for Risk Behavior Reduction, Tehran University of Medical Sciences, Tehran, Iran.*; 3 *Department of Reproductive Health, Faculty of Nursing and Midwifery, Tehran University of Medical Sciences, Tehran, Iran.*; 4 *Social Determinants of Health Research Center, Ahvaz Jundishapur University of Medical Sciences, Ahvaz, Iran.*; 5 *Trauma Research Center, Kashan University of Medical Sciences, Kashan, Iran.*; 6 *School of Medicine, Shahroud University of Medical Sciences, Shahroud, Iran.*; 7 *Reproductive Biotechnology Research Center, Avicenna Research Institute (ACECR), Tehran, Iran.*

**Keywords:** Psychometric properties, Sexual knowledge, Cross-cultural adaptation

## Abstract

**Background:**

Delivery of sexual health services rely on rigorous facts extracted from surveys, but often those facts cannot be available due to the lack of culturally-sensitive questionnaires.

**Objective:**

our aim was to show the validity and reliability of the Persian version of the Acquisition of Sexual Information Test (ASIT), a measure selected due to its assemblages with Iranian culture.

**Materials and Methods:**

Forward-backward procedure was applied to translate the questionnaire. Cross-sectional study was carried out and psychometric properties of the Iranian version were tested in a thirty sample of reproductive-age women. Face validity was assessed by qualitative and quantitative methods. Content validity was also assessed by calculating two quantitative indicators as content validity index (CVI) and content validity ratio (CVR). Reliability was assessed by test-retest analyses.

**Results:**

Impact score was 1.5, the majority of participants (83.3%) stated that the overall level of questionnaire was high but some of the questions were irrelevant to sexual knowledge. Many questions (90%) gained a CVR less than 0.56, and all of them gained CVIs lower than 0.7. Correlation in test-retest reliability was 0.85.

**Conclusion:**

sexual knowledge questionnaire seems to be culturally inappropriate for Iranian women. Although, we need survey data for assessing the evidence-based needs for sexual health and best practice, but the questions addressing various dimensions of sexuality must be culturally sensitive, comprehensive and appropriate. Our findings suggest that ASIT as a well-known measure should be used in Iranian population with caution.

## Introduction

Sexual health is an essential aspect of health that influences individuals across their lifespan in all ages (1). In study of Ronald et al. prevalence of sexual dysfunction was 20-30% and 40-45% in men and women respectively (2). Also one systematic review , reported a 35.2% prevalence of sexual dysfunction in Iran (2015) (3). Many studies consider sexual dysfunction as a possible cause of divorce (4-7). Sexual function is a complex process, resulting from interactions between vascular, neurological and hormonal factors, and is influenced by various determinants such as the biological characteristics, interpersonal relationships, traditions of the family and society, culture and religion(8). 

Sexual behavior and orientation are different among societies, due to differences in socio-cultural structure based on the theory of social constructionism (9). In many parts of the world there is no understanding of sexual health thus misunderstandings and socio -cultural barriers in sex education, result in low quality and quantity sexual health services (10). So the role of knowledge and awareness of sexuality is very important and can affect couples in all communicational and interactional aspects(11). Sexual knowledge is a set of data, information and awareness about sex and sexuality (12).

Sexual knowledge and attitude are related to sexual behavior, performance and health and several studies have found sexual knowledge and attitude associated with marital satisfaction (13-16). So that sexual knowledge, improves emotional relationship between them and lack of knowledge leads to marital conflict (16). Several studies by Masters and Johnson found that in many cases, sexual abnormalities are created by unawareness of sexual health system (17). Many other studies have shown that the cause of many sexual problems is low sexual knowledge. Lack of sexual knowledge results in a person's vulnerability, and undesirable sexual function (10). Several studies have indicated that sex education can be effective in promoting sexual health (18-20).

Regarding the role of sexual knowledge and its effect on promotion of sexual health, it is important to assess it by sensitive, reliable and convenient instruments. One of such questionnaires is “Anne Hooper’s” that includes 15 questions about sexual issues and can be used for both sexes. Although this questionnaire has been used in some studies in Iran (15, 21, 22), there are several socio-cultural considerations about the application of the measure and the same questionnaires. So, recently, some Iranian scholars tried to design tools for assessing sexual knowledge and attitude adopted with the socio-cultural context of Iran (13, 23). 

Another available questionnaire in this field is the “Acquisition of sexual Information Test” designed by Dusek, Monge and Lawless in 1977. Iranian researchers in sexology have emphasized the need for high quality scientific data on sexuality dimensions among adults, particularly with cultural considerations. Best practice and delivering sexual health services rely on rigorous facts extracted from surveys, but often those facts cannot be available due to the lack of culturally-sensitive questionnaires. 

So, the objective of this study was to assess the psychometric properties of the questionnaire in evaluating sexual knowledge.

## Materials and methods

This study was cross-sectional conducted on reproductive-age, married, healthy and sexually active women referred to Golabchi health care center affiliated to Kashan University of Medical Sciences, Kashan, Iran during June to October 2016. 

**Acquisition of Sexual Information Test**

Acquisition of sexual Information Test is a specific and self-report questionnaire developed by Dusek *et al* in 1977 (24). It consists of 50 items divided into five dimensions as venereal disease (items 50, 48, 33, 32, 29, and 5), contraception method, sexual relationship and reproduction (items 43, 39, 36, 16, 13), male biological aspects (items 45, 27, 21, 19, 14, 1), and female biological aspects (items 13, 16, 36, 40, 49). The remaining 26 items assess knowledge of various general aspects of human development and evolution (items 4, 5, 6, 15, 17, 18, 20, 22, 23, 24, 26, 28, 30, 31, 35, 37, 38, 41, 42, 44, 46, 47). The correct response for each item is indicated at the end of the test. 

Validity and reliability of the original research of the questionnaire were acceptable (24). This questionnaire has been used by several researchers such as Monge *et al* descriptive study (23). After gaining permission from the author (Professor Dusek), the forward-backward process was applied to translate from English into Persian based on the Brislin model. This model is one of the most reliable methods for translation that the process of translating of questionnaire is done in back and forth between origin and target languages (9). 

This model allows identifying errors and solving them during the translation process at once. Two independent professionals translated the questionnaire into Persian. Then one of the authors and translators compared Persian versions and produced a single Persian provisional version and temporary Persian version was extracted in this step. In third step, two other English experts translated the Persian version back into English. Finally the two questionnaire versions were revised and merged and a temporary English version was obtained. At the last step a temporary English version of the questionnaire was compared with the original version and necessary reforms were applied in order to create maximum coordination between the two versions and the final version of the questionnaire was developed.

**Statistical analysis**

Psychometric properties of the questionnaire were assessed by several statistical tests as follows:

**Validity**

Content validity**:** In this stage we used qualitative and quantitative methods (9). In this way questionnaire was given to 15 health professionals including reproductive health specialists, sexologist and health education experts. In qualitative stage, most of the experts who assessed the qualitative content validity stated that some items, especially those in the dimension of human evolution, have not enough adaptation to the Iranian context. In the quantitative stage, we used two indicators: the content validity index (CVI) and the content validity ratio (CVR) (9). About CVR, we used experts comments. For calculating this index the experts rate each item as “essential”, “useful but not essential”, or “not essential”.

Given that 12 experts of the 15 invited ones to the experts panel evaluated content validity of the tool, according to the Lawshe table, the minimum acceptable value for CVR was considered 0.56. To evaluate content validity, we used Waltz and Bausell index (12). CVI assesses the relevancy, simplicity and clarity criteria base on four item Likert scale. Given the number of members of expert panel, the minimum acceptable value for CVI was considered 0.79.

Face validity of the questionnaire was assessed by qualitative and quantitative methods (9). To assess the qualitative face validity, we received comments from target sample. In this stage, 20 women with convenience sampling (9) admitted to Golabchi health care center affiliated to Kashan University of Medical Sciences, Kashan, Iran were asked to assess difficulty, or obscurity in perception of the questions. Inclusion criteria were being Iranian, married, healthy, sexually active and in reproductive age (15-45 years). 

In the quantitative stage, we used the impact score (9). So, participants determined the importance of each item in a likert scale from 1 (not important) to 5 (very important). Impact score of those items equal or more than 1.5 was considered acceptable (9). Then impact scores were calculated by the following formula (9): 

Impact score= importance × Frequency (%) 

**Reliability**

Test-retest reliability was conducted to assess the questionnaire stability. Thirty participants completed the questionnaire twice in two-week intervals. A correlation higher than 0.8 was considered acceptable.

**Ethical consideration**

The ethics committee of medical sciences of Shahroud University approved the study with ethic code: IR.SHMU.REC.2015.44. 

## Results

Most of the participants (49.6%) were in age range of 26 to 35 years, duration of their marriage was less than 5 years (30.6%), had high school education (38.8%) and were housewives (80.2%).

**Validity**

**Content validity**

In the qualitative phase 10 experts (83.3%) stated that the overall difficulty level of the questions for the target population was high and simpler questions should be considered for this purpose. So, the question is not consistent with client education and they stated some of the questions were irrelevant to sexual knowledge such as those regarding sexual self-concept, social issues and human evolution. One expert (8.3%) noted that the questionnaire is composed of a large number of negative questions. None of the experts agreed with the questions of human evolution and believed those were not necessary to sexual knowledge. Other criteria such as grammar and wording were found to be appropriate. The result of quantitative content validity showed that mean CVI was 0.39 and CVR mean was -0.02. CVR and CVI of all questions are shown in table I. According to table, the majority (90%) of the questions gained a CVR less than 0.56, and also the CVI in all of the questions was less than 0.7. The least amounts of CVR and CVI belonged to sexuality relationship, pregnancy-related questions, human evolution, most of the questions related to sexually transmitted diseases, contraception methods and biological aspects of women and men.

**Face validity**

In the qualitative face validity, some participants expressed that they had difficulty in understanding the items (question 10, 15, 43), therefore obscurity of these questions were revised. So self-concept was written in parenthesis "self-concept is the picture and understanding of individual about themselves that make up in their minds." (Question 10) and we used the word “acquired” instead of “get” (question 15) and also “Vabaran [Farsi word for efferent]” instead “vas deferens”. Quantitative face validity was examined by calculating the impact score. This index was equal or greater than 1.5 (ranging from 1.5 to 3.2) for all questions. Therefore all items were preserved for the following steps.

**Reliability**

Correlation in test-retest reliability was 0.85 that was considered to be acceptable.

**Table I:** Content validity index (CVI) and the content validity ratio (CVR) in each domain

**Domains of questionnaire **	**Question number**	**CVI**	**CVR**
Sexually transmitted diseases	5, 29, 32, 33, 48, 50	0.4	0.125
Methods of contraception, sexual relations and fertility	2, 7, 8, 25, 34, 39, 42	0.43	-0.18
Male sexual biological aspects	1, 14, 19, 21, 27, 45	0.44	0.23
Female sexual biological aspects	13, 16, 36, 40, 49	0.56	0.6
General aspects of human evolution	3, 4, 5, 6, 9, 10, 11, 12, 13, 15, 17, 18, 20, 22, 23, 24, 26, 28, 30, 31, 35, 37, 38, 41, 42, 44, 46, 47	0.27	-0.4

## Discussion

Results from the present study indicated some considerable challenges in the cultural adaptation of the Persian version of Acquisition of Sexual Information Test. Regardless of our assumptions at the beginning of the study about selecting the Acquisition of Sexual Information Test, the Persian version of sexual knowledge questionnaire seems to be weak according to cultural adaptation in the Iranian context. The term of “cross-cultural adaptation” is defined as a process that includes both language and cultural adaptation in preparing a scale for use in another community (25). 

So, in this process, equality of concepts in original and target community is very important (26). Many questionnaires are designed in a country and are used in other countries with different cultures, while cross-cultural adaptation is not considered. The process of cultural adaptation can be broken down into three steps: (a) translation; (b) cross-cultural adaptation; and (c) verifying the psychometric properties of the instrument in the target population. Translation process needs to be addressed at three levels: semantic, technical, and conceptual. During this process, understandable translated version for the target population must be achieved (27). If the cross-cultural adaptation process is neglected, the findings will be incorrect (28). This is a complex process that methodologically requires high accuracy (25).

According to the cultural adaptation, we experienced some challenges in psychometric properties of the Persian version of the Acquisition of Sexual Information Test due to problems in concept translation and cross-cultural adaptation. Sex education programs focus on biological, social and psychological aspects of sex and emphasize that all of these aspects should be regarded (17). Although in the present study, the experts opposed the necessity of questions related to human evolution, psychological and social questions and stated those kinds of questions are of higher social levels.

In quantitative content validity, specialists stated that difficulty level of questions is too high for the study population and should be designed simpler. Considering few numbers of studies about sexual knowledge questionnaires in Iran, comparing our results with other studies seems to be difficult. But in a study by Besharat *et al*, though designed with a different approach, two variables were considered including knowledge and attitude; the validity and reliability were found to be accurate. questionnaire of Besharat *et al* is approved through implementation simultaneous Glumbok Marital Status questionnaire, Romantic Relationship Scale and Mental Health Inventory but this questionnaire is a limited number of questions so that with only 15 questions measure sexual knowledge (13).

Finally, sexuality, and related issues as well as knowledge and attitude are strongly affected by several factors including biological, cultural, ethical, legal, historical, religious, and spiritual factors, which influence individual’s sexual self-understanding (9). The complexity of this area is superimposed to difficulty of the process of measurement, also translation and cultural adaptation of related questionnaires. Our findings suggest that ASIT as a well-known measure should be used in Iranian population cautiously. It must be re-validated with different adult populations than that of our study. 

Considering these issues, it is recommended that the process of adaptation should be done with more caution and in some situations development of contextual tool with high degrees of appropriateness toward socio-cultural norms of the target population is suggested. There was a significant limitation in our study which is the few number of sexual health experts for the panel, so we seeked help from other related professionals such as psychologists and reproductive health specialists.

## Conclusion

Asking cultural sensitive questions about sexuality allows researchers to draw a clear picture of the study population. The complexity of Iranian culture is expressed in intersections between sexuality related issues and gender schema. Accordingly, methodological considerations as well as cross-cultural adaptation are recommended. 

## Note

***This article is extracted from a Ph.D. Thesis. (Zahra Karimian)***
